# Approaches to Observe Anthropogenic Aerosol-Cloud Interactions

**DOI:** 10.1007/s40641-015-0028-0

**Published:** 2015-11-11

**Authors:** Johannes Quaas

**Affiliations:** Institute for Meteorology, Universität Leipzig, Stephanstr. 3, 04103 Leipzig, Germany

**Keywords:** Aerosol-cloud interactions, Radiative forcing, Observational constraint, Anthropogenic emissions, Cloud modification, Ship tracks, Hemispherical contrast, Weekly cycles, Pollution trends

## Abstract

Anthropogenic aerosol particles exert an—quantitatively very uncertain—effective radiative forcing due to aerosol-cloud interactions via an immediate altering of cloud albedo on the one hand and via rapid adjustments by alteration of cloud processes and by changes in thermodynamic profiles on the other hand. Large variability in cloud cover and properties and the therefore low signal-to-noise ratio for aerosol-induced perturbations hamper the identification of effects in observations. Six approaches are discussed as a means to isolate the impact of anthropogenic aerosol on clouds from natural cloud variability to estimate or constrain the effective forcing. These are (i) intentional cloud modification, (ii) ship tracks, (iii) differences between the hemispheres, (iv) trace gases, (v) weekly cycles and (vi) trends. Ship track analysis is recommendable for detailed process understanding, and the analysis of weekly cycles and long-term trends is most promising to derive estimates or constraints on the effective radiative forcing.

## Introduction

The radiative forcing anthropogenic aerosol particles exert when they serve as cloud condensation nuclei (CCNs) and ice nucleating particles (INPs) and thus modify cloud properties: the forcing due to aerosol-cloud interactions is the most uncertain one among the forcings of anthropogenic climate change [1]. An enhanced CCN concentration leads to larger concentrations of cloud droplets in liquid water clouds. It also leads to larger concentrations of ice crystals in ice clouds when they form by homogeneous freezing of haze particles in suitable conditions [2]. Even for constant cloud water path, when the average particle sizes are reduced, this enhances the overall scattering cross section and cloud albedo is larger than without the additional anthropogenic aerosol [3]. In case of heterogeneous freezing in ice clouds, in contrast, additional INP may lead to fewer, larger ice crystals, reducing cirrus albedo [4].

The shift in particle size spectra impacts cloud microphysical and dynamical processes. Growth of cloud particles via collision and coalescence depends on the relative velocity of the particles in an air parcel, mostly determined by the fall velocity. Since fall velocity is a non-linear function of particle size, a change in size spectra alters this process and, hence, the precipitation formation. It has been postulated that the delay in precipitation formation may lead to larger cloud cover [5], but this is disputed [6–9]. Cloud depth and cloud water path are expected to increase [10]. Since fallout of water from the liquid phase is reduced, in deep convective clouds, the anvil created from detrainment might become thicker in a polluted environment [11]. Other processes may lead to decreases in cloud radiative effects. For liquid water clouds, smaller particles more readily evaporate due to the larger surface-to-volume ratio, so an increase in CCN may also lead to less, thinner clouds [6]. Also, the cloud-top entrainment rate is, besides its dependency on the thermodynamic profiles, a function of precipitation rate for shallow, stratiform clouds [12]. Depending on the humidity above the cloud layer, a reduction in precipitation may thus lead to more entrainment of dry air and, subsequently, a reduction in cloudiness [13] and liquid water path [14]. For ice- and mixed-phase clouds, INP might lead to more rapid glaciation of a cloud and shorter lifetime [15]. A phase change from liquid to ice may be favoured by additional INP, leading to smaller albedo [16, 17].

Absorption of sunlight and subsequent heating of the atmosphere might lead to more or less cloudiness, depending on the altitude of the aerosol relative to the cloud [18–21]. The various processes interact in a dynamical environment, often leading to a buffering of the initial perturbation [9, 22].

The concept of the effective radiative forcing [23, 24] considers the radiative forcing itself that is introduced virtually immediately with an altered aerosol concentration due to the change in cloud albedo, with all other state variables unchanged, but also fast cloud adjustments that occur on short time scales, namely faster than changes in the large-scale circulation or surface temperatures [25–27]. The effects described in the first paragraph above constitute the radiative forcing due to aerosol-cloud interactions [1] (formerly known as first aerosol indirect effects). The effects described in the second paragraph refer to the adjustments to the aerosol-cloud interactions. The third paragraph finally introduced the adjustments to the aerosol-radiation interactions (formerly known as semi-direct effect). Besides these cloud microphysical and microdynamical adjustments, also the surface temperatures on continents and, subsequently, the thermodynamic profiles react, leading to further adjustments [20, 28].

There is a sound theoretical basis for several of the concepts of possible effects of anthropogenic aerosols on clouds and radiation described above at the level of individual microphysical processes. The relevant relationships, e.g. between cloud droplet concentration and cloud albedo, have been observed from aircraft [29–31]. Statistical relationships between aerosol and cloud or radiation quantities as retrieved from satellite data support the expected sensitivities of cloud albedo with respect to aerosol perturbations. These relationships have been useful to constrain processes of aerosol-cloud interactions in climate models [32–37] to the degree of yielding a radiative forcing estimate or constraint [38–40]. However, these kinds of statistics themselves do not allow to infer conclusions about anthropogenic perturbations. In the context of this paper, the full anthropogenic perturbation with respect to a natural-only reference is generally used to avoid the definition of the small anthropogenic perturbations even of the 1850 aerosol concentrations [41, 42].

The fact that the uncertainty in radiative forcing is so large is, thus, not primarily due to a lack in process understanding or in process observation. It rather is due to the fact that (a) the *anthropogenic* contribution to the perturbation of clouds is uncertain and (b) the overall effect, involving all interacting processes, quantitatively is highly uncertain.

The aim of this review is to identify and discuss approaches by which the perturbation of clouds and, subsequently, radiation by anthropogenic aerosols can be *observed* in order to either directly quantify the radiative forcing or constrain it as simulated by climate models. I identified six methods that have been proposed to this end:

1. *Intentional cloud modification*: In field experiments and also at local scales semi-operationally, clouds have been seeded artificially with aerosol in weather modification or climate engineering experiments.
2. *Ship tracks* are evident manifestations of an anthropogenic perturbation of clouds in the marine boundary layer.
3. *Hemispheric contrast*: Since anthropogenic emissions mostly take place in the Northern Hemisphere, a difference in cloud and radiation properties might be indicative of the anthropogenic perturbation.
4. *Trace gases*: While it is difficult to clearly distinguish anthropogenic from natural aerosol in measurements, some trace gases such as carbon monoxide can clearly be linked to combustion. Such tracers may mark air masses that are polluted and allow distinction from unperturbed ones. A related approach is to use chemistry transport models to identify anthropogenically influenced air masses.
5. *Weekly cycles*: Seven-day rhythms are highly unlikely due to natural variability and, thus, allow for a clear attribution to anthropogenic perturbations, if they can be identified with certainty.
6. *Trends*: There are long-term trends in anthropogenic aerosol emissions. To the extent that consistent cloud and radiation trends are observed, these may be attributed to the emission trends.

In the following, an overview is given about the state of the art and applicability of each of these six approaches.

## Intentional Cloud Modification by Aerosol

Since the 1940s, experimental studies have been conducted aiming at weather modification. The principal aim of these was to (i) reduce hail, (ii) reduce fog and, most prominently, (iii) enhance rainfall by (a) seeding mixed-phase clouds with ice nucleating particles in an attempt to exploit the Bergeron-Findeisen effect to produce precipitation [43], (b) seeding supercooled liquid clouds in an attempt to increase buoyancy exploiting the latent heat of freezing [44] or (c) seeding liquid water clouds with giant CCN to circumvent delays in the collision-coalescence process [45–47].

Despite very large efforts, however, results are inconclusive [48]. Even when exploiting a land-sea contrast and orography and, thus, a forced repetitive condition for precipitation, as done in long-term Israeli experiments, no clear conclusions could be drawn [49]. A recent aircraft experiment in seeding marine stratocumulus with large aerosol particles was able to demonstrate shifts in microphysical characteristics [50] but did not proceed to study cloud albedo nor were they able to assess cloud modifications at a large scale beyond the selected individual ship tracks. In a climate engineering field experiment, a smoke generator onboard a ship below a stratocumulus deck off the North American West coast was used to artificially create CCN. The zigzag track pattern was identified as a bright line in satellite imagery, and aircraft in situ observations allowed to document the cloud microphysical differences from the background [51]. However, only for a few cases was the detection possible.

Intentional cloud modification studies are potentially useful for an improved process understanding [52, 53]. However, they were not suitable for a constraint of the aerosol forcing due to their limited extent and the differences between the intentional and inadvertent emission of aerosols in terms of aerosol types, mixing state and spatio-temporal distributions.

## Ship Tracks

Ship tracks are bright lines detectable from satellites in marine boundary layer clouds [54] in suitable conditions [55, 56]. These lines are attributed to the emission of suitable CCN by ship exhaust [57] that alters cloud droplet concentrations and, subsequently, cloud albedo [58]. They may be particularly visible since the surrounding area tends to become less bright due to induced boundary layer dynamics [59]. In turn, the effect may become very large in cases where a transition between open and closed cellular structure of stratocumulus clouds may be triggered [60, 61]. Also in simulations with general circulation models, the global forcing attributable to ship emissions can yield, depending on the assumptions and large forcing estimates [62, 63].

However, the actual lines only account for 0.04 of the global area [64] and thus exert a very small radiative forcing on a global scale of estimated about −0.5 × 10^−3^ Wm ^−2^ [65]. A substantial diurnal cycle demonstrated from geostationary satellite data, with a maximum at the time of overpass of the polar-orbiting satellite from which this forcing was estimated, even suggests a smaller effect [66]. The effect of ship pollution beyond the narrow tracks is not distinguishable with statistical significance compared to the regions upwind the shipping routes [67]. This is due to the dilution of the aerosol that does spread around the ship track on the one hand and due to the large natural variability of clouds on the other hand [63, 68].

Nevertheless, ship tracks have proven very useful to identify and assess processes relevant for aerosol-cloud interactions [69–71], providing, for example, evidence for a shift in response between liquid- and mixed-phase clouds [72•]. The analysis of ship tracks showed that the response of cloud properties and cloud albedo depends on the cloud regime and differs between coupled and uncoupled boundary layers [73] and open and closed cells [74]. In summary, ship tracks have proven to provide a good environment to examine processes of aerosol-cloud interactions but are not useful to constrain the aerosol effective forcing at large scales.

While contrails from aircraft exhaust are also clear manifestations of an anthropogenic perturbation of cloudiness [75, 76], much of the perturbation is due to humidity perturbation rather than aerosol perturbations [77]. Contrails are, thus, not as suitable to assess aerosol-cloud interactions.

## Hemispheric Contrast

Most anthropogenic aerosol sources are located in the Northern Hemisphere. The short lifetime of the aerosol confines their effects also mostly to the Northern Hemisphere, in which aerosol-cloud interaction effects are expected to be much larger [78]. Anthropogenic SO_2_ is, to a dominating extent, emitted in the Northern Hemisphere, with 98 vs. 6 Tg S year^−1^ [79]. Moderate Resolution Imaging Spectroradiometer (MODIS) retrievals of the aerosol optical depth (AOD) show a distinct hemispheric contrast even over ocean, with an AOD of about 50 % larger in the Northern Hemisphere [80]. For MODIS-retrieved fine-mode AOD, the difference over the oceans is 0.094 vs. 0.061 [81]. Hemispheric differences were also found for droplet sizes. Annual (1987 and 1988) average droplet effective radii from the Advanced Very High Resolution Radiometer (AVHRR) on board the NOAA-9 and NOAA-10 satellites show a contrast of 11.0 μm in the Northern Hemisphere vs. 11.7 μm in the Southern Hemisphere [82]. MODIS shows a difference of 12.4 vs. 13.0 μm in droplet effective radii [81]. However, for the optical depth of liquid water clouds, the results are inconclusive: AVHRR shows a cloud optical depth of 6.6 in the Northern Hemisphere and 7.4 in the Southern Hemisphere [82]. This result was found both over land and ocean. Cloud optical depth from MODIS, in turn, was found to be slightly larger in the Northern Hemisphere over ocean at 12.6 vs. 12.1 in the Northern Hemisphere vs. the Southern Hemisphere. The contrast, however, was much smaller than predicted by a chemistry transport model [81]. Cloud albedo is also larger in the Southern Hemisphere than in the Northern Hemisphere for mid to high latitudes [79].

These results imply that the clouds between the hemispheres differ not only due to the differences in aerosol but also due to differences in the large-scale circulation and in the land-ocean distribution. The differences in the aerosol are not completely attributable to anthropogenic activities, since some of the major deserts are in the Northern Hemisphere and biomass burning differences are also not solely due to anthropogenic combustion. The fact that cloud albedo is not significantly larger, or even smaller, in the Northern Hemisphere is an indication that the aerosol is not a first-order factor for cloud properties [79]. The hemispheric difference in aerosol emissions and the observed warming in both hemispheres consequently also allows to infer an upper bound in the aerosol forcing [83•].

In summary, the hemispheric contrast is useful because it allows to leverage many observations. However, the convolution of many different effects hampers the analysis of individual effects. As such, only broad constraints on the aerosol-cloud interactions are possible.

## Trace Gases

The possibilities to observe aerosols from satellites or ground-based remote sensing in cloudy regions are limited. In contrast, trace gases may be observed also in cloudy skies. Also, aerosols are emitted from both natural and anthropogenic sources so that a clear identification of the anthropogenic component is difficult. In turn, some trace gases such as CO are clearly attributable to combustion. While this includes natural fires, in many situations, it can be attributed to anthropogenic activity. CO and aerosol concentrations are tightly related near the source regions [84, 85] as long as the concentrations are more determined by the source strength than by the lifetime. CO lifetime is longer than the aerosol lifetime, since CO is less efficiently scavenged and deposited compared to the aerosol. The influence of oxidants on both, CO and aerosol, complicates the relationship [86]. Despite these limitations, relationships between CO concentrations and cloud properties have been found in observations that are promising for process analysis. Elevated concentrations of CO have been demonstrated to coincide with increased cloud droplet number concentration and decreased cloud droplet effective radii as well as with increased cloud optical depth [87].

A related approach is to combine observations with chemistry transport modelling in weather forecast mode. The model results allow the identification of air masses subject to pollution, and observations of cloud and radiation in polluted conditions can be compared to less polluted or unpolluted ones [88–90].

In conclusion, retrievals of trace gases from satellites may help to identify anthropogenic pollution. However, ambiguity remains especially due to the differences in lifetime and sinks. Also, models may help to identify anthropogenic aerosol. A challenge in either case is to distinguish the effect due the anthropogenic emissions from other perturbations that are convolved with the pollution such as different origins and histories of air masses.

## Weekly Cycles

An unambiguous 7-day cycle is hardly attributable to natural variability. Trace gases like NO_2_ show distinct minima on Sundays over North America and Europe, on Fridays over the Middle East and on Saturdays over Israel and no clear minimum over China [91]. Early studies of the weekly cycle were intended to demonstrate a greenhouse effect and found cooler temperatures on weekends over the Northern Hemisphere [92]. Weekly cycles in precipitation have been found with a minimum coincident with the pollutant minimum [93] or pollutant maximum [94]. To date, weekly cycles for many meteorological parameters are inconclusive and differ among the different studies [95•]. The attempt to detect and attribute weekly cycles in cloud properties such as cloud fraction and liquid water path, radiation fluxes or meteorological parameters such as surface temperature to anthropogenic aerosols fails except for the basic parameters such as aerosol optical depth and cloud droplet number concentration [96]. A problem is the challenge of assessing statistical significance: The occurrence of a single maximum and minimum each, among just seven instances, is rather likely, and high confidence levels are required [95•].

The analysis of weekly cycles, nevertheless, is a particularly promising avenue for a constraint and quantification of the effects of anthropogenic aerosols. Combination of modelling and observations may allow for a clear attribution of weekly cycles in particular quantities to anthropogenic aerosol. The relative importance of the weekly cycle in emission with respect to total emissions may allow an estimation of the anthropogenic radiative forcing, should a weekly cycle in radiation be identifiable, by scaling the weekly amplitude to the total emission strength.

## Trends

Anthropogenic aerosol emissions increased substantially since pre-industrial times and have subsequently decreased in some regions such as Europe in the past few decades [97]. At the same time, solar radiation at the surface has changed by −7 Wm^−2^ globally between 1961 and 1990 [98]. Over North America, a larger decreasing trend was found for cloudy compared to clear skies [98]. In turn, since a few decades, the trends are reversed in many regions from this ‘dimming’, leading to a ‘brightening’, or increases in surface solar radiation both in clear and cloudy skies [99]. This increasing trend coincides, over Europe, with a decline of fog and haze and an increase in visibility [100]. Particularly strong increasing trends in aerosol emissions occurred over China. These coincided with decreases in visibility and increases in aerosol optical depth, but also in meteorological parameters [101]. Climate models show that the trend in all-sky surface solar radiation due to aerosol emission reductions is correlated to the effective radiative forcing by anthropogenic aerosol, allowing for a constraint on the simulated aerosol forcing [102•]. Trends in other meteorological quantities may be related to aerosol effects as far as the models show a clear link between observable trends in such quantities and anthropogenic aerosol emissions. In this regard, trends in the diurnal temperature range [103], monsoon precipitation [28, 104, 105], Sahel drought [106] or the North Atlantic oscillation [107] have been attributed to anthropogenic aerosol emissions.

Besides long-term trends, there are occasionally also abrupt changes in anthropogenic emissions. In the period 11–14 September 2001, all commercial air traffic was banned over North America. This has been exploited to detect a change in the diurnal temperature range possibly attributable to the lack in contrails [108, 109]. During the Olympic Games in Beijing, emission controls led to substantial transient reductions in air pollution [110, 111]. Fireworks lead to occasional substantial enhancements in anthropogenic pollution [112, 113]. Such short, singular events are, however, difficult to exploit for a systematic process analysis and are not suitable for a forcing assessment.

Trends are thus, despite the ambiguity, especially valuable for a constraint on the aerosol forcing and for studies attributing observed climate change aspects to aerosols. They are not as suited for an improved process understanding concerning the interaction of aerosols with cloud microphysical and microdynamical processes.

## Conclusions

The large uncertainty in anthropogenic aerosol forcing that hampers quantitative understanding and prediction of current climate change urgently needs to be reduced. Ideal for this would be an estimate from observations, or else a constraint of the simulated forcing, based on observations. So far, process-oriented metrics (statistical relationships between relevant quantities such as between aerosol and cloud droplet concentration, cloud droplet concentration, albedo, etc.) were used for this goal. However, since a multitude of fast adjustments is acting and effects may be buffered, the actual observation of the perturbation of clouds and the radiation budget unequivocally attributable to anthropogenic aerosol is highly desirable. The challenge is due to the large natural variability in clouds and their strong impact on radiation. Even in the global annual mean, the cloud radiative effect is about −50 Wm^−2^ [114], while the aerosol forcing is of the order of 2 % of this.

Six approaches to this end are identified and discussed in this review. All of them are useful, but as summarised in Table 1, two of them (ship tracks and intentional weather modification) are only for an improved process understanding, not an actual estimate of, or constraint on, the forcing. This is due to the small scales they are applicable to. The analysis of the hemispheric contrast is difficult since perturbations clearly attributable to aerosols cannot be disentangled from other influences. Nevertheless, important constraints can be derived from hemispheric differences. Using trace gases as markers of anthropogenic pollution, or using modelling to identify pollution, is useful for both, process analyses and forcing assessment, but only in a limited scope close to source regions. The assessment of weekly cycles and trends emerges as the most powerful avenue to infer relevant, quantitative information about the aerosol forcing, but also to a more limited extent, process assessment.

**Table 1:** Methods to observe the anthropogenic aerosol effect and possible applications for a radiative forcing estimate or for improved process-level understanding of the effect of anthropogenic aerosol on clouds and radiation

Method	Forcing estimate	Process understanding
Weather modification	-	x
Ship tracks	-	x
Hemispheric contrast	l	-
Trace gases	l	l
Weekly cycles	x	l
Trends	x	l

‘-’ stands for little use, ‘l’ limited applicability, and ‘x’ for promising applicability

Obtaining a significant signal-to-noise ratio for both, trends and weekly cycles, requires long time series of consistent measurements. Thus, the extension of current observational records, both ground based and space borne, is a prerequisite for increasingly convincing assessments.

Most useful are studies that combine the observational assessments with model sensitivity studies in order to clearly attribute effects in observations to anthropogenic emissions and particular processes by which these affect clouds and the radiation budget. This requires models that realistically represent the relevant processes and their interactions. A successful forcing estimate or constraint thus calls for an iterative approach: improved process understanding from observations analysis (e.g. ship track analysis) and subsequent model improvement, and forcing estimates and constraints from observations in combination with model sensitivity studies, best from weekly cycle or trend analysis.
